# Three New Humulane Sesquiterpenes from Cultures of the Fungus *Antrodiella albocinnamomea*

**DOI:** 10.1007/s13659-014-0032-4

**Published:** 2014-07-17

**Authors:** Zi-Ming Chen, Qiong-Ying Fan, Xia Yin, Xiao-Yan Yang, Zheng-Hui Li, Tao Feng, Ji-Kai Liu

**Affiliations:** 1 State Key Laboratory of Phytochemistry and Plant Resources in West China, Kunming Institute of Botany, Chinese Academy of Sciences, 132# Lanhei Road, Heilongtan, Kunming, 650201 Yunnan People’s Republic of China; 2 College of Life Science, Hebei Normal University, Shijiazhuang, 050024 People’s Republic of China

**Keywords:** *Antrodiella albocinnamomea*, Humulane-type sesquiterpenes, Protein tyrosine phosphatase inhibitory activity

## Abstract

Three new humulane-type sesquiterpenes, antrodols A–C (**1**–**3**), were isolated from cultures of the fungus *Antrodiella albocinnamomea*. Their structures were elucidated on the basis of extensive spectroscopic analysis. Antrodols A–C (**1**–**3**) are first examples of humulane-type sesquiterpenes isolated from cultures of higher fungi, and antrodol A (**1**) was the first report of humulane-type sesquiterpene with a methyl rearranged at C-3. All compounds were evaluated in the enzyme inhibition assay against two protein-tyrosine phosphatases (PTPs): MEG2 and PTP1Bc.

**Electronic supplementary material:**

The online version of this article (doi:10.1007/s13659-014-0032-4) contains supplementary material, which is available to authorized users.

## Introduction

**Fig. 1:**
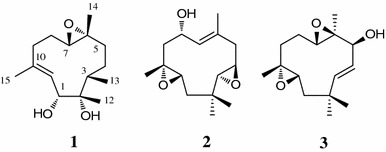
Structures of compounds **1**–**3**

Higher fungi have been proven to be rich sources of secondary metabolites with unusual structures as well as interesting biological activities [1]. Over the last 10 years, our main research has been focusing on bioactive secondary metabolites from the untapped resources of higher fungi collected in China [2–4]. As a continuation of our studies on biologically active natural products from higher fungi, chemical investigation on fermentation broths of *Antrodiella albocinnamomea* has resulted in the isolation of three new humulane-type sesquiterpenes, antrodols A–C (**1**–**3**). Fungal sesquiterpenes formed via the humulane-protoilludane biosynthetic pathway are characteristic chemical markers for the subdivision Basidiomycota. The largest group of sesquiterpenes belonging to the classes of lactaranes, secolactaranes, marasmanes, isolactaranes, norlactaranes, and caryophyllanes were believed to be derived from humulane [5, 6]. However, only a few humulane sesquiterpenes were reported from higher fungi previously [7–10]. To the best of our knowledge, antrodols A–C (**1**–**3**) were the first examples of humulane-type sesquiterpenes isolated from the culture broths of higher fungus, and antrodol A (**1**) was the first report of humulane-type sesquiterpene with a methyl rearranged at C-3 (Fig. 1). The inhibitory activities of the isolated compounds against two protein-tyrosine phosphatases (PTPs): MEG2 and PTP1Bc were evaluated. Herein we report the isolation, structure elucidation and biological activities of these three new humulane-type sesquiterpenes.

## Results and Discussion

Compound **1** was obtained as colorless oil. Its molecular formula was determined to be C_15_H_26_O_3_ on the basis of HREIMS ([M]^+^ at *m/z*, 254.1887), with three degrees of unsaturation. The IR spectrum showed absorption bands at 3440 cm^−1^ and 1635 cm^−1^, indicating the presence of hydroxyl group and double bond. The ^13^C NMR spectrum exhibited 15 carbon signals, including one trisubstituted double bond resonances at *δ*_C_ 138.2 and 125.7, four oxygen-bearing carbons at *δ*_C_ 62.1, 63.1, 71.6 and 75.4, and four methyl signals at *δ*_C_ 14.5, 15.6, 18.0 and 19.3 (Table 1) (Fig. 1)

**Table 1:** ^1^H and ^13^C NMR spectroscopic data for compounds **1**–**3** (*J* in Hz)

No.	1		2		3	
	*δ*_C_	*δ*_H_	*δ*_C_	*δ*_H_	*δ*_C_	*δ*_H_
1	71.6 d	4.16 dd (9.9, 5.3)	39.5, t	1.54 dd (15.1, 9.0)	39.2, t	1.40 dd (14.2, 9.6)
				1.61 d (14.8)		1.51 d (14.2)
2	75.4 s		33.9, s		35.9, s	
3	41.6 d	1.56 dq (13.2, 6.6)	64.2, d	2.31 d (2.4)	140.3, d	5.49 d (15.9)
4	26.4 t	0.94 (m)	55.8, d	3.22 (m)	129.0, d	5.53 dd (15.9, 7.7)
		1.12 (m)				
5	38.0 t	2.00 dd (13.5, 9.4)	40.1, t	1.29 dt (11.3, 5.7)	81.8, d	3.55 dd (7.7, 3.5)
		0.80 dd (13.6, 8.6)		2.35 dd (12.4, 5.1)		
6	62.1 s		135.2, s		66.0, s	
7	63.1 d	2.64 dd (11.0, 1.6)	130.0, d	5.29 dt (9.4, 1.4)	58.8, d	2.70 dd (9.9, 5.1)
8	24.7 t	1.85 (m)	65.6, d	4.47 (m)	25.6, t	2.15 (m)
		1.49 (m)				1.35 (m)
9	37.9 t	2.26 (m)	48.7, t	1.14 (m)	35.5, t	2.00 ddd (13.6, 5.7, 2.3)
		2.31 td (12.7, 5.0)		2.35 dd (12.4, 5.1)		1.10 dd (15.9, 5.2)
10	138.2 s		60.3, s		60.2, s	
11	125.7 d	5.45 d (9.9)	61.7, d	2.75 d (8.5)	64.4, d	2.45 d (9.6)
12	19.3 q	1.05 (s)	18.3, q	0.86 (s)	23.5, q	1.21 (s)
13	14.5 q	0.92 d (6.6)	29.8, q	1.03 (s)	30.8, q	1.07 (s)
14	18.0 q	1.15 (s)	19.8, q	1.78 (s)	10.8, q	1.26 (s)
15	15.6 q	1.71 (s)	17.4, q	1.15 (s)	16.7, q	1.27 (s)
1-OH		3.87 d (5.4)				
2-OH		3.23 (s)				
5-OH						4.34 d (3.6)
8-OH				3.80 d (3.1)		

600 and 150 MHz, in acetone-*d*_6_

**Fig. 2:**
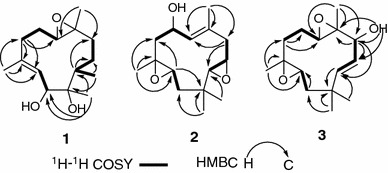
Key ^1^H-^1^H COSY and HMBC correlations of **1**–**3**

**Fig. 3:**
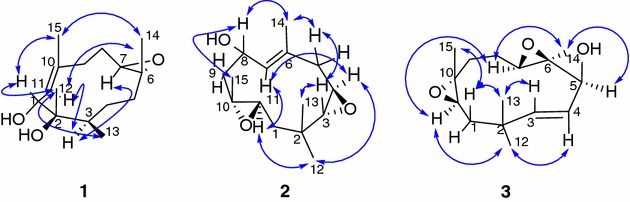
Key ROESY correlations of **1**–**3**

In the ^1^H–^1^H COSY spectrum, correlations established coupling relationships of Me-13/H-3/H_2_-4/H_2_-5, H-7/H_2_-8/H_2_-9, and H-1/H-11, as shown with bold lines in Fig. 2. The location of the functional group and the assembly of compound **1** were done by HMBC data. The HMBC spectrum showed correlations of H-12/C-1, C-2, C-3 and H-3/C-2, C-1, indicating a partial structure comprised C-1–C-2–C-3 with a methyl (C-12) attached at C-2. The HMBC correlations of H-14/C-5, C-6, C-7 and H-7/C-5, C-6 suggested a moiety of C-5–C-6–C-7 with a methyl (C-14) attached at C-6. The HMBC correlations of H-15/C-9, C-10, C-11 suggested that a methyl group (C-15) linked at C-10, forming an 11-membered carbocyclic ring of compound **1**. The epoxide ring at C-6 and C-7 was deduced from appropriate carbon chemical shifts at *δ*_C_ 62.1 and 63.1, as well as MS data analysis and HMBC correlations. The above evidence allowed the elucidation of the planar structure of **1** to be a humulane-type sesquiterpene with a methyl rearranged at C-3. The relative configuration of **1** was determined by an ROESY experiment. The 10*E* configuration was determined by the carbon resonances of C-10 (*δ* 138.2) and C-15 (*δ* 15.6), which were similar to related compound [8]. This assumption was also supported by the ROESY correlation between Me-15 and H-1, while the ROESY correlation between Me-15 and H-11 can not be detected. In the ROESY experiment, the observed ROESY correlations between Me-15 and Me-14, H-1; between Me-14 and Me-12; between Me-12 and Me-13 indicated that H-1, Me-12, Me-13, Me-14 and Me-15 were in the same side (assigned as *β*-orientation). The ROESY correlations between H-3 and H-11, H-7 revealed that H-3, H-7 and H-11 were in the *α*-orientation. Based on the above data, we deduced the favorable configuration of **1** as shown in Fig. 3, which was compatible with that offered by molecular modeling. So compound **1** was elucidated as 6,7-epoxy-10*E*-humulen-1,2-diol, and has been named antrodol A.

Compound **2** was obtained as an amorphous solid and its molecular formula was determined to be C_15_H_24_O_3_ by the HREIMS at *m/z* 275.1622 [M]^+^(calcd for C_15_H_24_O_3_Na, 275.1623), suggesting four degrees of unsaturation. Its IR data exhibited absorption bands for a hydroxy group (3462 cm^−1^) and an olefinic bond (1640 cm^−1^). The 1D NMR data of **2** strongly resemble to those of (2*R*,3*R*,5*S*)-2,3-epoxy-6,9-humuladien-5-ol-8-one(**4**) (Table 1) [11]. The key difference was that carbon resonances for CH=CHC=O moiety in **4** (*δ*_C_ 160.0, 129.3 and 200.9) were upfield in **2** (*δ*_C_ 64.2, 55.8 and 40.1), suggesting the double bond in **4** was replaced by an epoxide ring in **2** and the carbonyl was changed to a methylene. In the HMBC spectrum (Fig. 2), the correlations from Me-13 to C-3 and Me-14 to C-5 confirmed this change. The relative configuration of **2** was determined by an ROESY experiment. The ROESY correlations between H-1*β* and Me-13, Me-15; H-8 and Me-15; H-8 and Me-14, H-5*β* and H-8, H-3 revealed that H-3, H-8, Me-13, Me-14 and Me-15 were in the *β*-orientation. The ROESY correlations between Me-12 and H-4, H-11; H-4 and H-7 indicated that H-4, H-7, H-11 and Me-12 were in the opposite orientation (*α*). From these data, compound **2** was determined to be 3,4:10,11-diepoxy-6*E*-humulen-8*α*-ol, named antrodol B.

Compound **3** was assigned the same molecular formula as that of **2** (C_15_H_24_O_3_) by HREIMS at *m/z* 275.1622 [M]^+^ (calcd for C_15_H_24_O_3_Na, 275.1623). The IR and 1D NMR data were extremely similar to those of **2**, indicating that compound **3** was also a humulane-type sesquiterpene. Compared to **2**, the main differences were below: (I) the epoxide ring at C-3 and C-4 in **2** was replaced by a double bond in **3**; (II) the ∆^6^ olefinic bond was replaced by an epoxide ring; (III) the hydroxy group was placed at C-5 in **3** rather than at C-8 in **2**. Those assignments were deduced by 2D NMR data analysis. In the ^1^H-^1^H COSY spectrum, the cross-peaks of H-3/H-4/H-5 indicated a CH=CH–CH moiety in **3**; the hydroxy was at C-5 due to the downfield chemical shift at *δ* 81.8; the epoxide ring at C-6 and C-7 was deduced from the characteristic ^13^C NMR signals at *δ* 66.0 and 58.8. The above assignments were corresponded with the HMBC correlations from Me-13 to C-3, from 5-OH to C-4, C-5 and C-6, from Me-14 to C-5, C-6 and C-7 as shown in Fig. 2. The relative configuration of **3** was established by an ROESY experiment. In the ROESY spectrum, the ROESY correlations between Me-12 and H-4 and H-11; H-5, H-7, H-11 and Me-14 indicated that H-4, H-5, H-7, H-11, Me-12 and Me-14 were in the same side (assigned as *α*-orientation) while the ROESY correlations between Me-13 and H-3, H-1*β*; and H-1*β* and Me-15 demonstrated that H-3, Me-13 and Me-15 were in the *β*-orientation. The *E*-geometry of ∆^3^-double bond was further determined by the large coupling constant of ^3^*J*_H-3/H-4_ (15.9 Hz). Consequently, compound **3** was determined to be 6,7:10,11-diepoxy-3*E*-humulen-5*β*-ol, and named antrodol C.

The biological activities of antrodols A–C (**1**–**3**) were evaluated in the enzyme inhibition assay against three PTPs: MEG2 and PTP1Bc. As summarized in Table 2, antrodols A (**1**) was demonstrated to have moderate inhibitory activities against protein-tyrosine phosphatase MEG2 and PTP1Bc with IC_50_ values of 8.0 and 10.0 μg/mL, respectively. Antrodol C (**3**) was showed mild inhibitory activities against protein-tyrosine phosphatase PTP1Bc with IC_50_ values of 15.1 μg/mL.

**Table 2:** The inhibitory activity of compounds **1**–**3** against protein-tyrosine phosphatases, IC_50_ (μg/mL)

Compounds	MEG2	PTP1Bc
**1**	8.0	10.0
**2**	NA	NA
**3**	NA	15.1
Ursolic acid^**a**^	0.8	1.2

*NA* no activity

^**a**^Positive control

## Experimental Part

### General Experimental Procedures

Optical rotations were taken on a Horiba SEAP-300 polarimeter. UV spectra were obtained on a Hitachi UV 210A spectrophotometer. IR spectra were measured on a Bio-Rad FTS-135 spectrometer with KBr pellets. 1D and 2D NMR spectra were recorded on a Bruker Avance III 600 MHz spectrometer (Karlsruhe, Germany). ESIMS and HREIMS were recorded with an API QSTAR Pulsar I spectrometer. Preparative MPLC was performed on a Buchi apparatus equipped with Buchi fraction collector C-660, Buchi pump module C-605 and manager C-615. Column chromatography was performed on silica gel (200–300 mesh; Qingdao Marine Chemical Co. Ltd., Qingdao, People’s Republic of China) and Sephadex LH-20 for chromatography was purchased from Amersham Biosciences. Fractions were monitored by TLC and spots were visualized by heating silica gel plates sprayed with 10 % H_2_SO_4_ in EtOH.

### Fungal Material and Fermentation

*Antrodiella albocinnamomea* was provided by Prof. Yu-Cheng Dai, Institute of Microbiology, Beijing Forestry University, and fermented by Mr. Zheng-Hui Li, Kunming Institute of Botany. A voucher specimen is deposited in the Institute of Microbiology, Beijing Forestry University. The culture medium consisted of glucose (5 %), peptone from porcine meat (0.15 %), yeast powder (0.5 %), KH_2_PO_4_ (0.05 %), and MgSO_4_ (0.05 %).The fermentation was carried out at 27 °C for 360 h with aeration at 0.4 vvm (air volume/culture volume/minute) and agitation at 150 rpm on a 100 L fermenter.

### Extraction and Isolation

The fermented whole broth (70 L) was filtered through cheesecloth to separate into supernatant and mycelia. The former was concentrated under reduced pressure to about a quarter of the original volume and then extracted three times with ethyl acetate (20 L×3) at room temperature, and the organic solvent was evaporated to dryness under reduced pressure to afford a brown crude extract. The mycelia was extracted three times with acetone (10 L×3). The acetone solution was concentrated under reduced pressure to afford an aqueous solution. The aqueous solution was extracted three times with ethyl acetate (3 L×3), and the ethyl acetate solution was evaporated to dryness under vacuum to obtain the crude extract of the mycelia. Both of the extracts were combined for further purification (45 g).

The combined crude extract was then fractionated by silica gel column chromatography (CC) eluted with a gradient of petroleum ether–acetone (100:0–0:100) to obtain eight fractions. Fraction 3 was eluted with petroleum ether–acetone (3:1), was then purified into five subfractions (3A–3E) by MPLC using MeOH/H_2_O as eluent. Fraction 3B was then separated by Sephadex LH-20 eluting with acetone to give **1** (2.6 mg). Fraction 4 was eluted with petroleum ether–acetone (2:1), was further purified by MPLC using MeOH/H_2_O as eluent to afford seven subfractions (4A–4G). 4C was then subjected to Sephadex LH-20 (acetone) and silica gel CC (petroleum ether–ethyl acetate, 7:3) to yield **2** (6.2 mg) and **3** (5.8 mg).

### Antrodol A (**1**)

Colorless oil; [*α*]_D_^25^  + 16.46 (*c* 0.23, MeOH); UV (MeOH) *λ*_max_ (log *ε*) 202.8 (3.73) nm; IR (KBr) *ν*_max_ 3440, 2925, 1635 cm^−1^; ^1^H and ^13^C NMR spectra data see Table 1; ESIMS (positive) *m/z*: 277 [M+Na]^+^; HREIMS: *m/z* 254.1887 [M]^+^(calcd for C_15_H_26_O_3_, 254.1882).

### Antrodol B (**2**)

An amorphous solid; [*α*]_D_^25^ + 75.20, (*c* 0.31, MeOH); UV (MeOH) *λ*_max_ (log *ε*) 202.4 (3.53) nm; IR (KBr) *ν*_max_ 3441, 2958, 2928 1631, 1387 cm^−1^; ^1^H and ^13^C NMR spectra data see Table 1; ESIMS (positive) *m/z* 275 [M+Na]^+^; HREIMS: *m/z* 275.1599 [M]^+^(calcd for C_15_H_24_O_3_Na, 275.1623).

### Antrodol C (**3**)

An amorphous solid; [*α*]_D_^25^ – 84.18, (*c* 0.29, MeOH); UV (MeOH) *λ*_max_ (log *ε*) 201.2 (3.35), 229.2 (2.40) nm; IR (KBr) *ν*_max_ 3462, 2962, 2928 1640, 1462, 1451, 1392 cm^−1^; ^1^H and ^13^C NMR spectra data see Table 1; ESIMS (positive) *m/z* 275 [M+Na]^+^; HREIMS: *m/z* 275.1622 [M]^+^(calcd for C_15_H_24_O_3_Na, 275.1623).

### Biological Activity Assay

Human MEG2 and PIP1Bc with an N-terminal 6× His-tag were recombinantly expressed in *E. coli* and purified by Ni–NTA affinity chromatography [12–14]. The enzymatic assay was carried out at room temperature in 96-well plates. After the assay buffer which contained 100 mM Hepes (pH 6.0), 5 mM DTT 0.015 % Brij-35 and PTPase (20 ng PTP1B, 10 ng MEG2per well) was incubated with tested compounds for 15 min, the reaction was initiated by addition of the substrate p-nitrophenol phosphate (pNPP, Sigma, P4744, St. Louis, USA) at a final concentration of 2 mM. The activity of PTPase-catalyzed hydrolysis of pNPP was determined by measuring the amount of *p*-nitrophenol and the absorbance at 405 nm was recorded as the amount of *p*-nitrophenol. The IC_50_ value was determined by the non-linear curve fitting of the percentage inhibition versus inhibitor concentration plot. Ursolic acid (Sigma, U6753, St. Louis, USA) was used for the positive control [15]. All the assays were carried out in triplicate and the average results were presented.

## Electronic supplementary material

Below is the link to the electronic supplementary material.

Supplementary material 1 (DOCX 1607 kb)
