# Supplementary material for: Three New Humulane Sesquiterpenes from Cultures of the Fungus Antrodiella albocinnamomea
Source: Nat Prod Bioprospect. 2014 Jul 17;4(4):207–11. doi: 10.1007/s13659-014-0032-4 (PMC4111878; doi:10.1007/s13659-014-0032-4)
Supplement: Supplementary file 1 — Supplementary material 1 (DOCX 1607 kb) [file 13659_2014_32_MOESM1_ESM.docx]

# New humulane-type Sesquiterpenenes from cultures of the fungus *Antrodiella albocinnamomea*

Zi-Ming Chen ^a^, Qiong-Ying Fan ^b^, Xia Yin ^a^, Xiao-Yan Yan^a^, Zheng-Hui Li ^a^, Tao Feng ^a^, Ji-Kai Liu^a^[[1]](#footnote-2)^*^,

*^a^ State Key Laboratory of Phytochemistry and Plant Resources in West China, Kunming Institute of Botany, Chinese Academy of Sciences, Kunming 650201, People’s Republic of China.*

*^b^College of Life Science, Hebei Normal University, Shijiazhuang 050024, People’s Republic of China.*

**Contents**

Figure 1S‒8S: NMR and MS spectra of antrodol A (**1**)……………………..p2S-5S

Figure 9S‒16S: NMR and MS spectra of antrodol B (**2**) ……….……………p6S-9S

Figure 17S‒21S: NMR and MS spectra of antrodol C (**3**) …………........…p10S-13S

Figure 1S. ^1^H NMR (600 MHz, acetone-*d*_6_) spectrum of antrodol A (**1**)


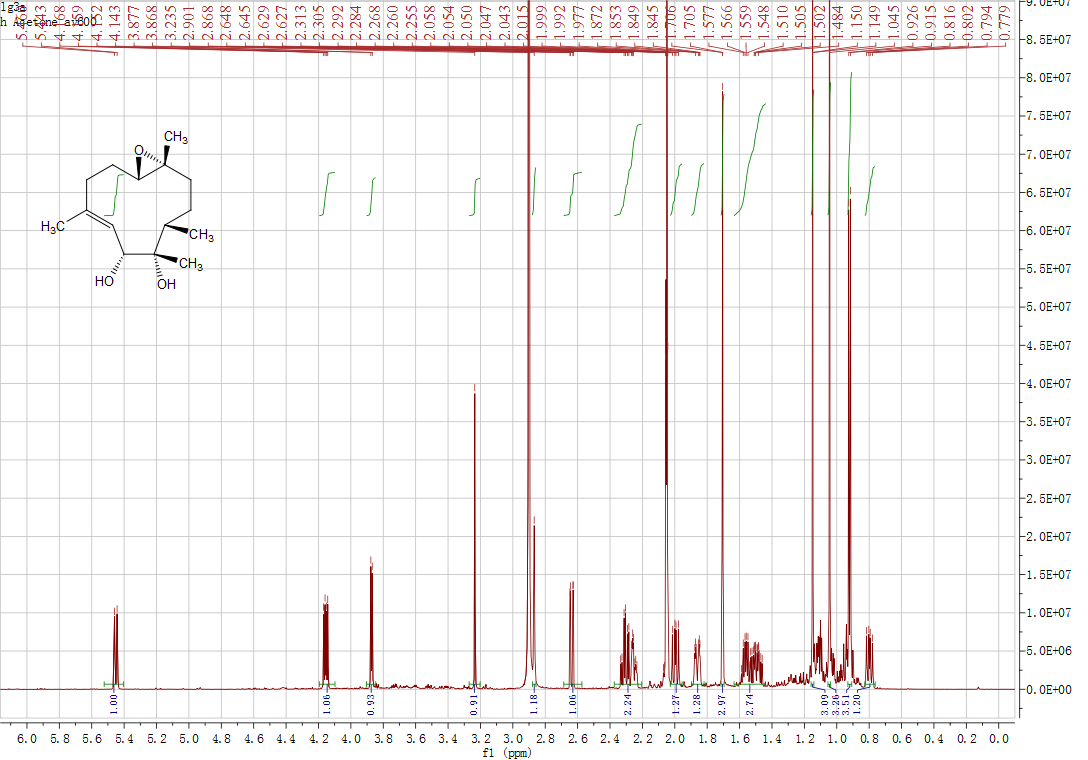


Figure 2S. ^13^C NMR and DEPT (150 MHz, acetone-*d*_6_) spectra of antrodol A (**1**)


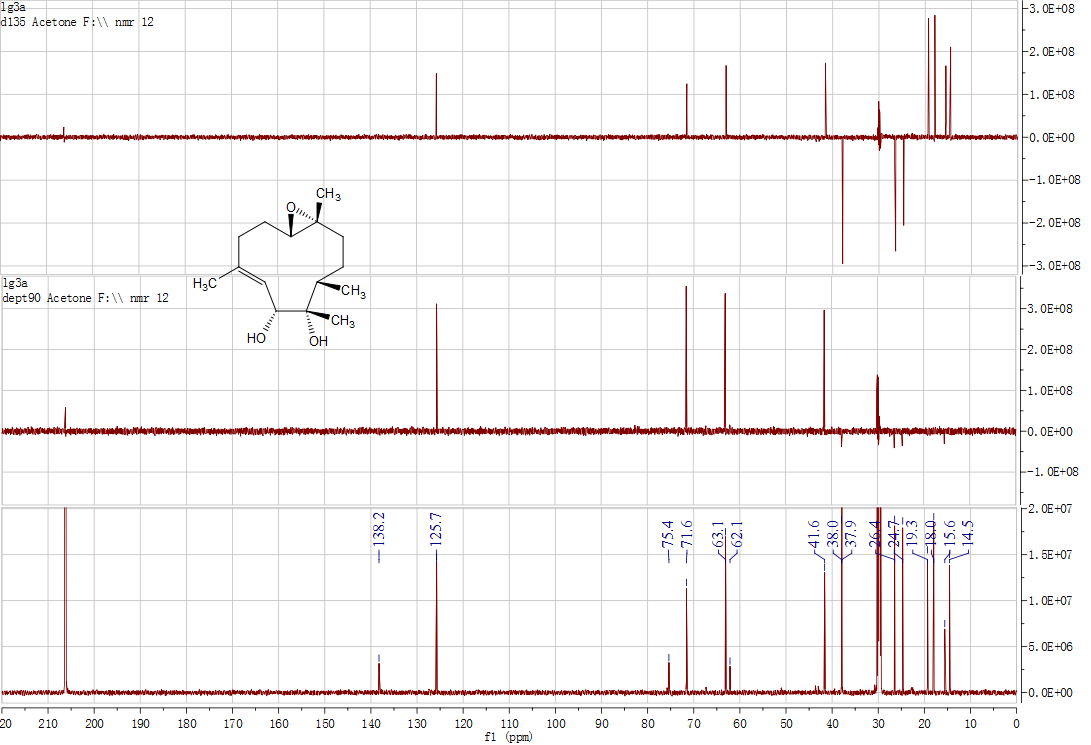


Figure 3S. ^1^H-^1^H COSY spectrum of antrodol A (**1**)


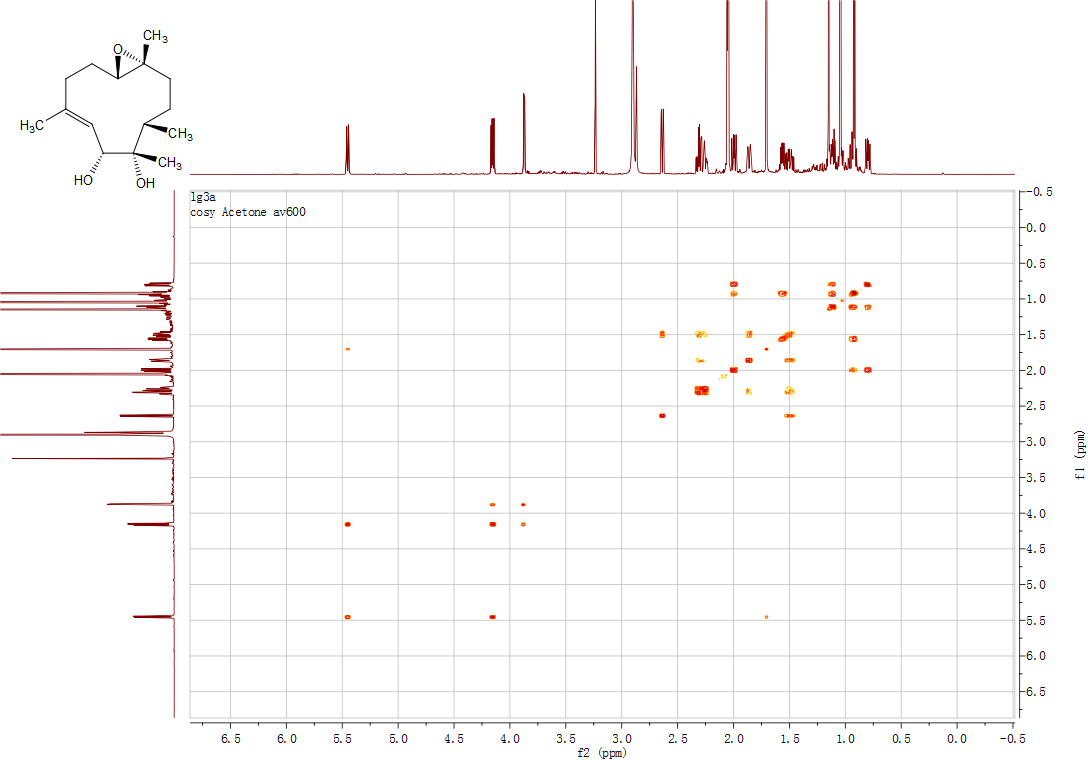


Figure 4S. HSQC spectrum of antrodol A (**1**)


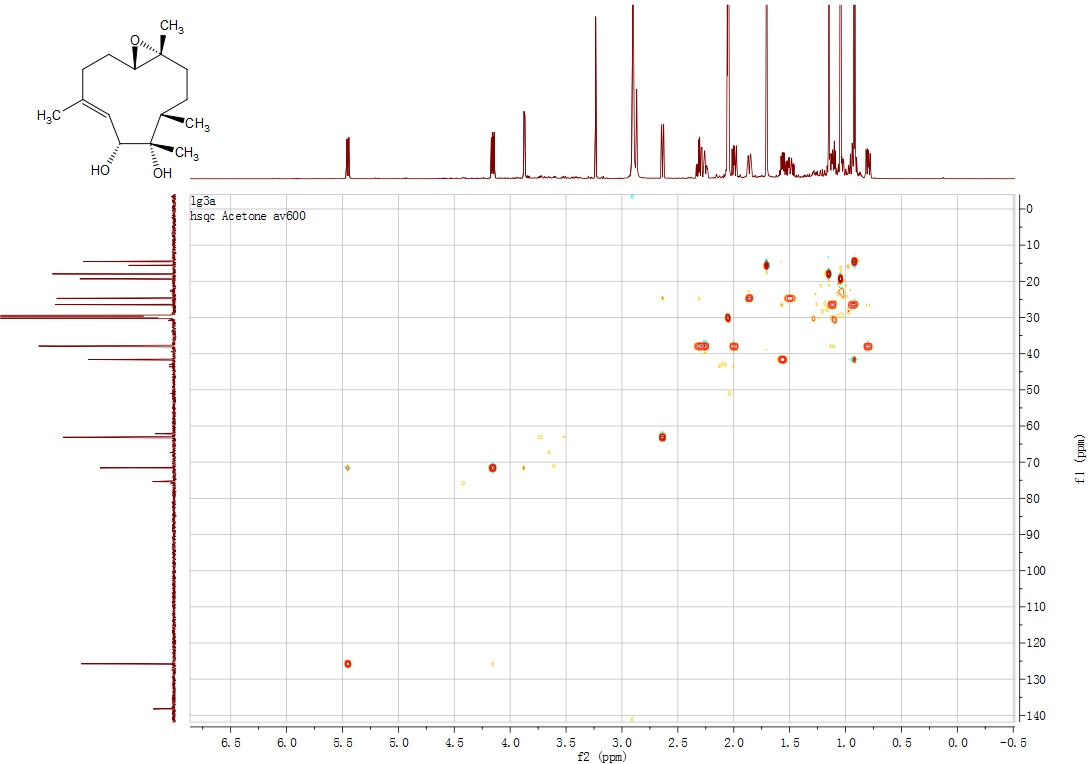


Figure 5S. HMBC spectrum of antrodol A (**1**)


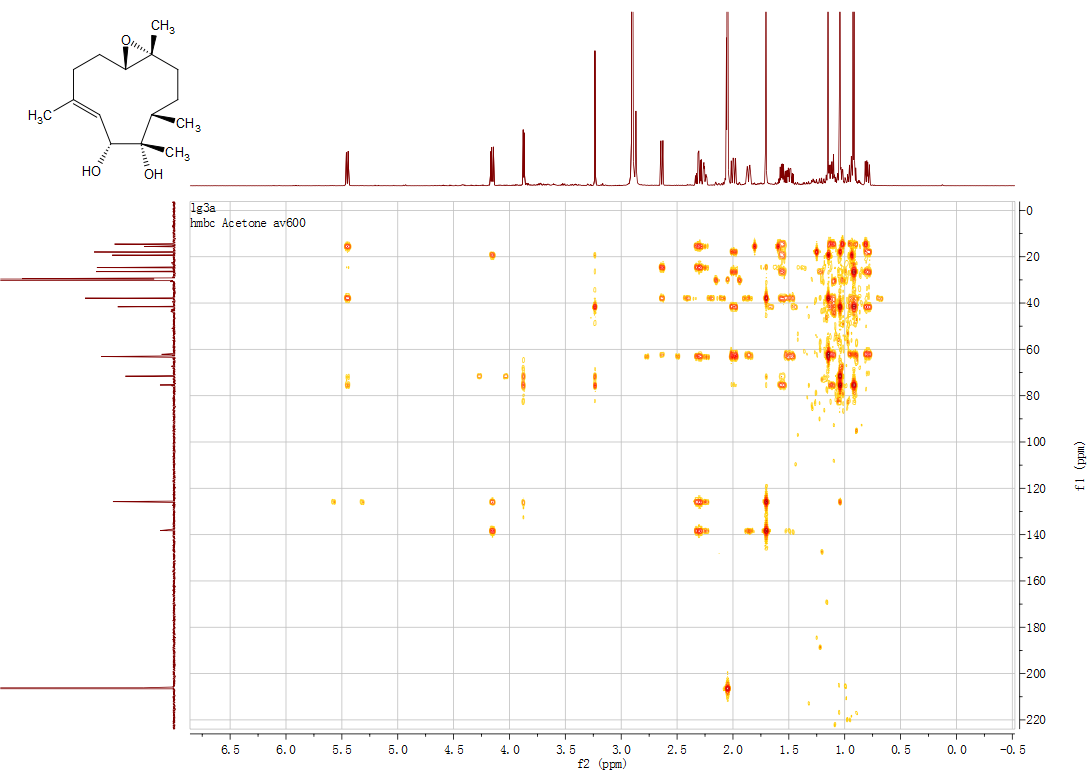


Figure 6S. ROESY spectrum of antrodol A (**1**)


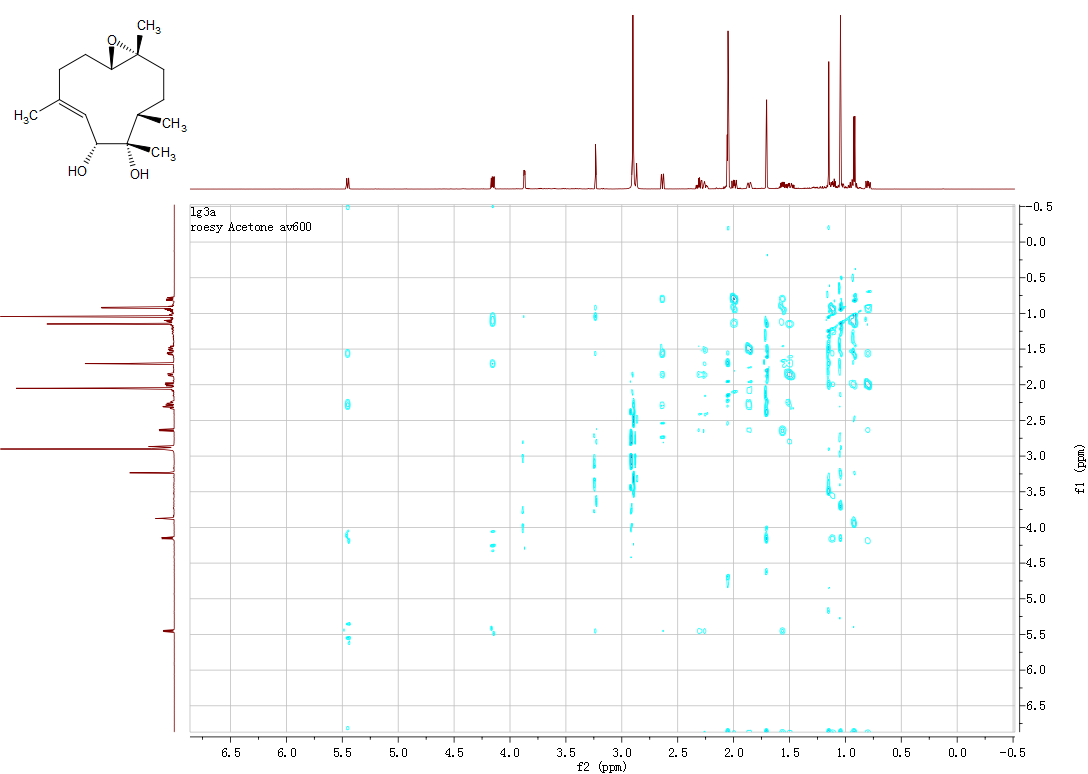


Figure 7S. HREIMS of antrodol A (**1**)


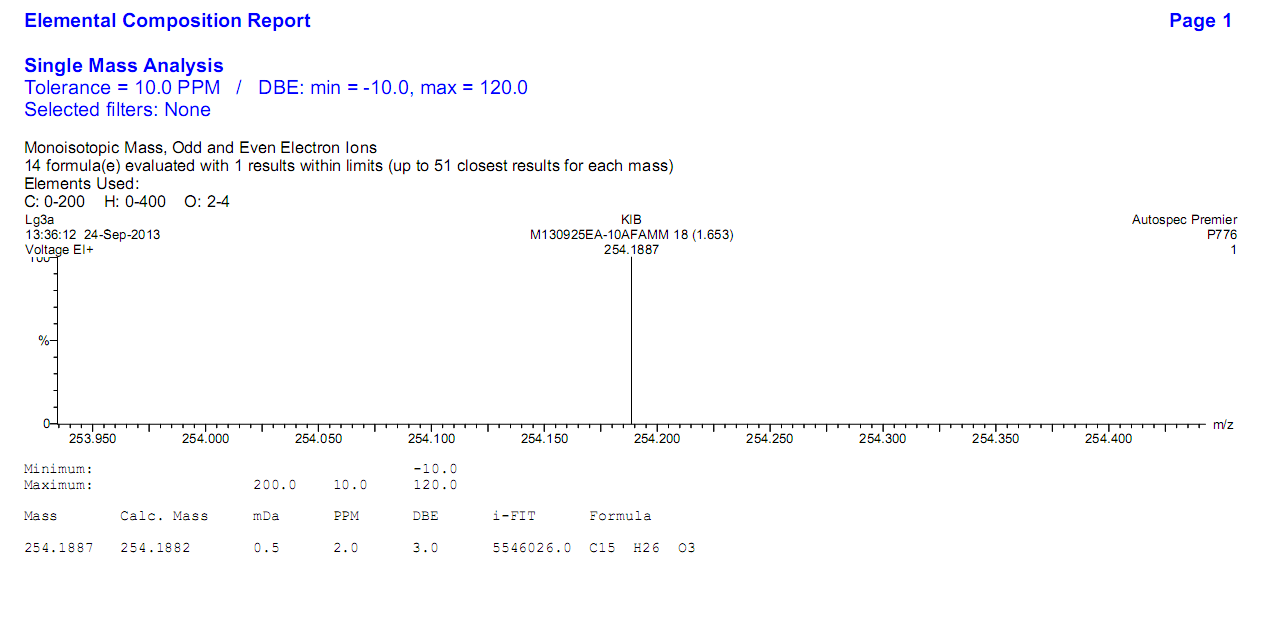


Figure 8S. ^1^H NMR (600 MHz, acetone-*d*_6_)spectrum of antrodolB (**2**)


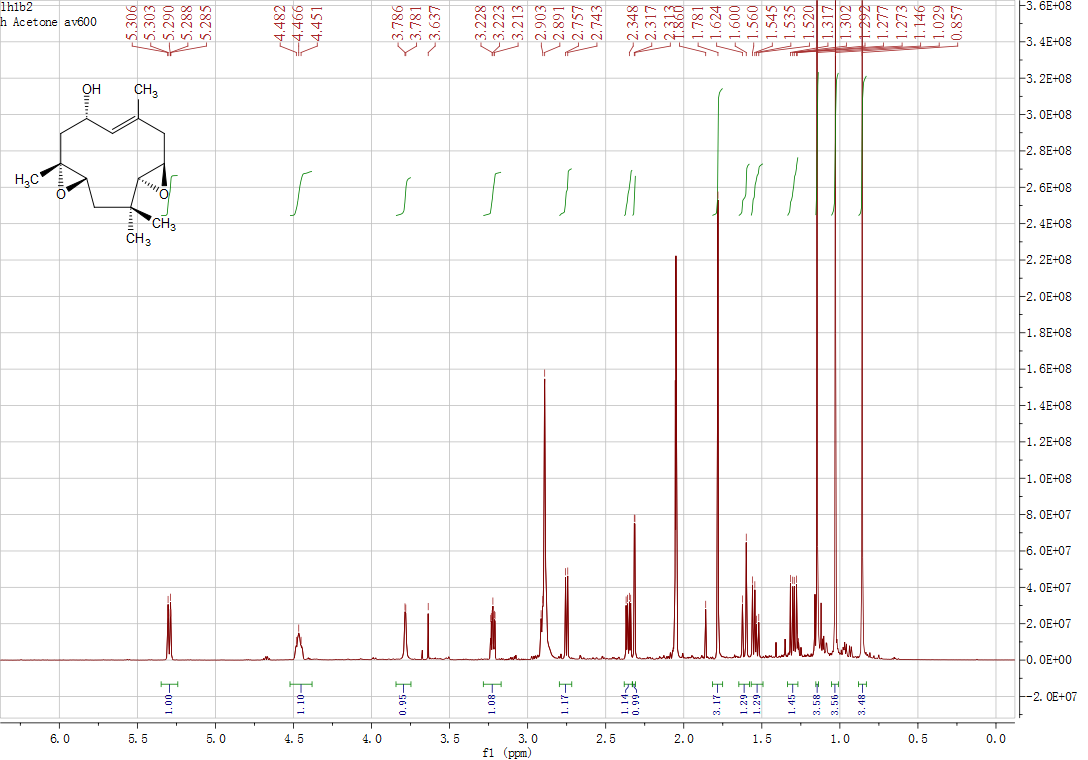


Figure 9S. ^13^ C NMR and DEPT (150 MHz, acetone-*d*_6_) spectra of antrodolB (**2**)

**
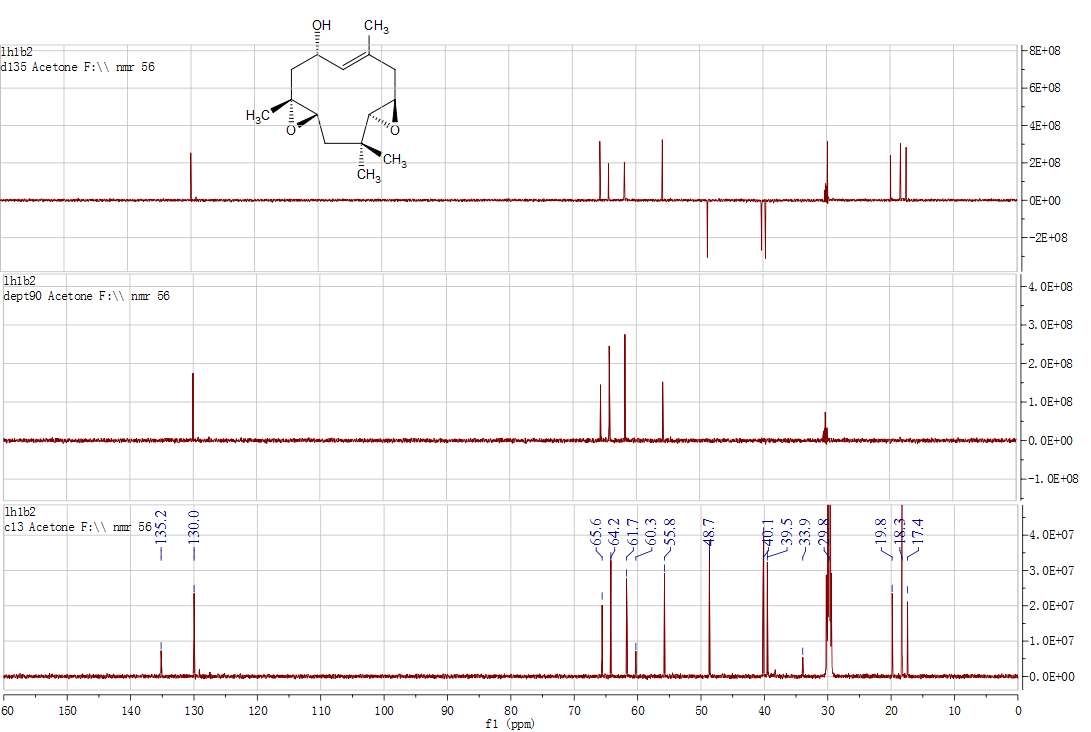
**

Figure 10S. ^1^H-^1^H COSY spectrum of antrodolB (**2**)


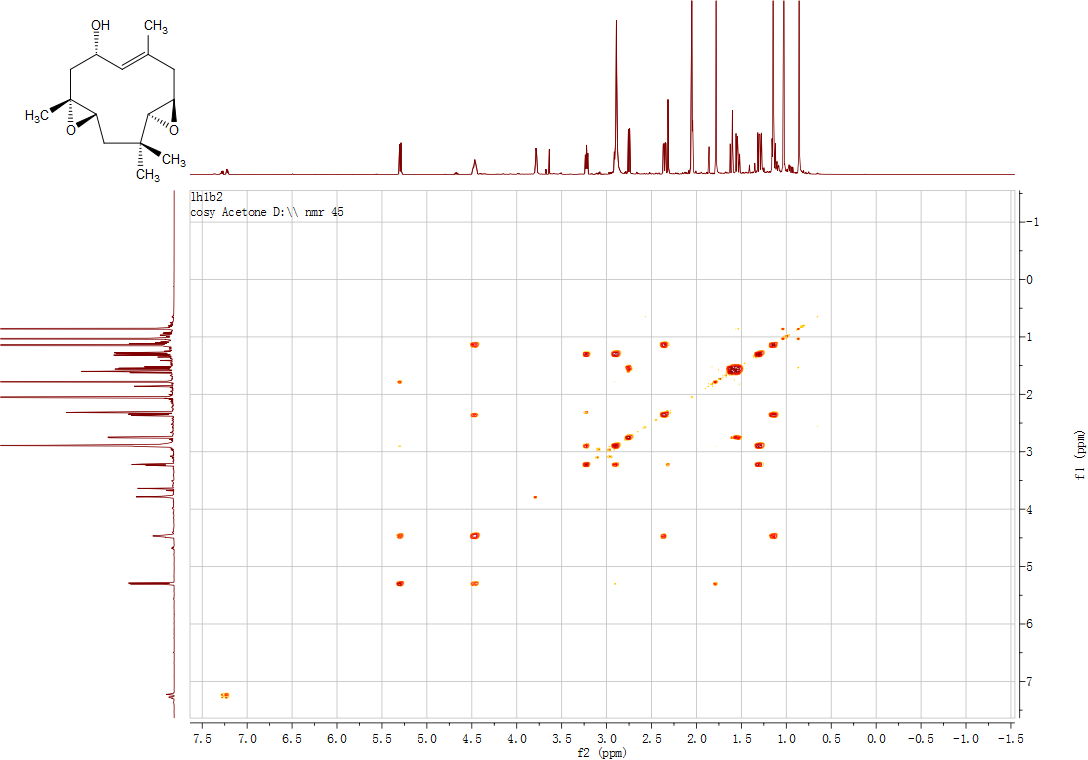


Figure 11S. HSQC spectrum of antrodolB (**2**)


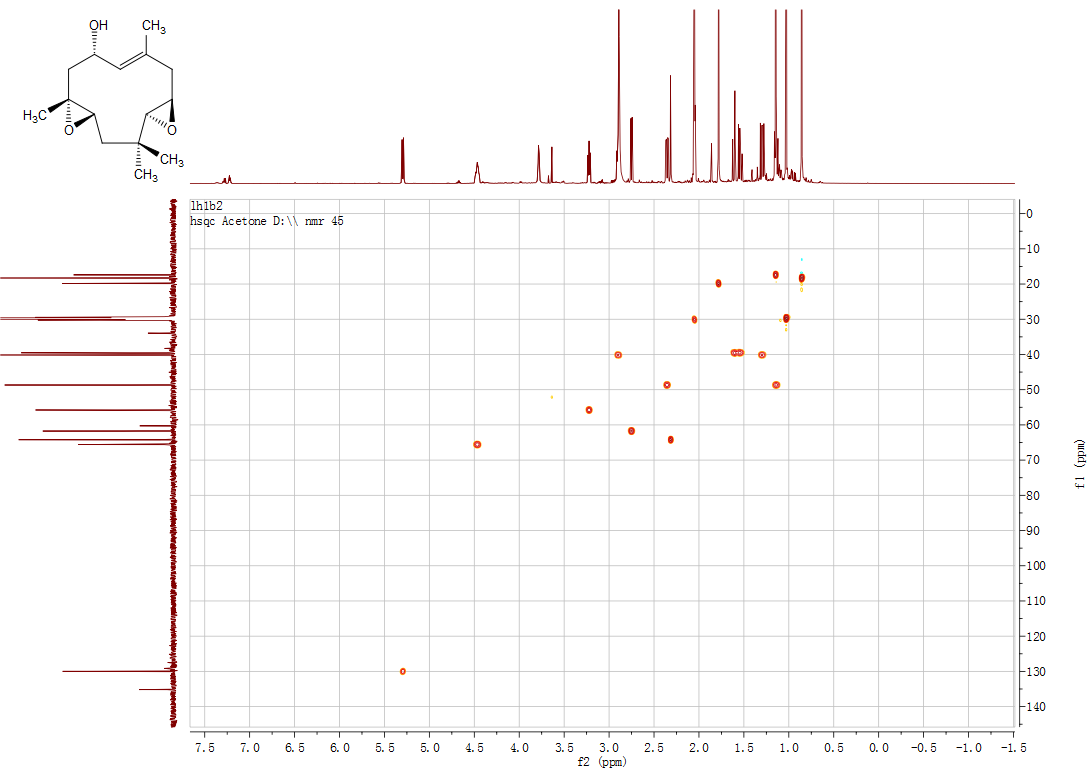


Figure 12S. HMBC spectrum of antrodolB (**2**)


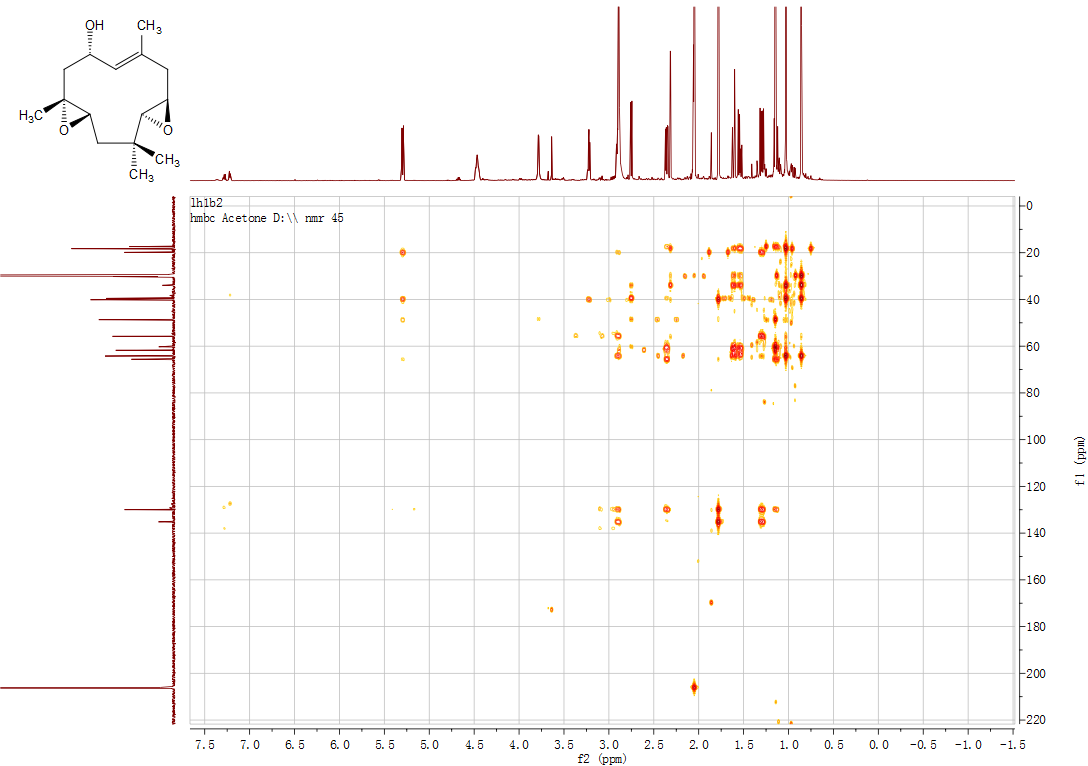


Figure 13S. ROESY spectrum of antrodolB (**2**)


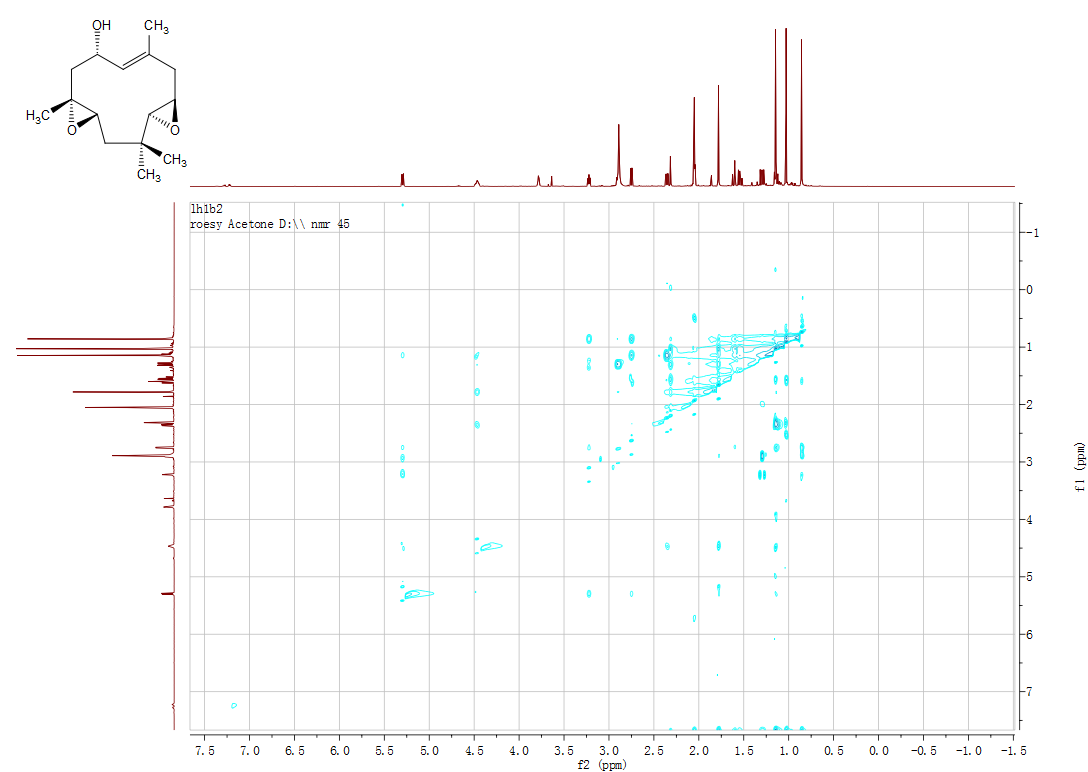


Figure 14S. HREIMS of antrodol B (**2**)


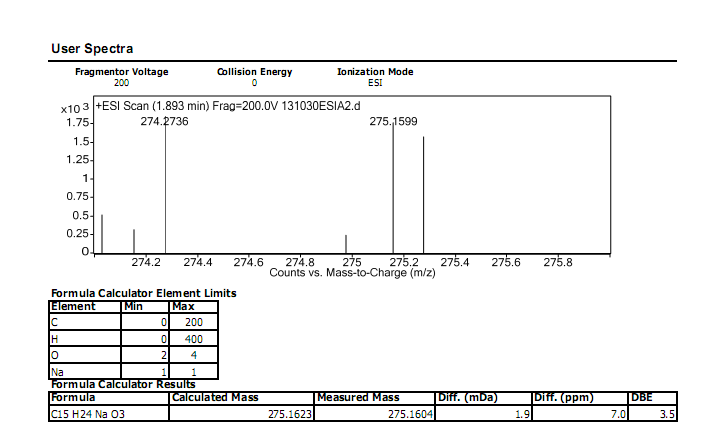
Figure 15S. ^1^H NMR (600 MHz, acetone-*d*_6_) spectrum of antrodol C (**3**)


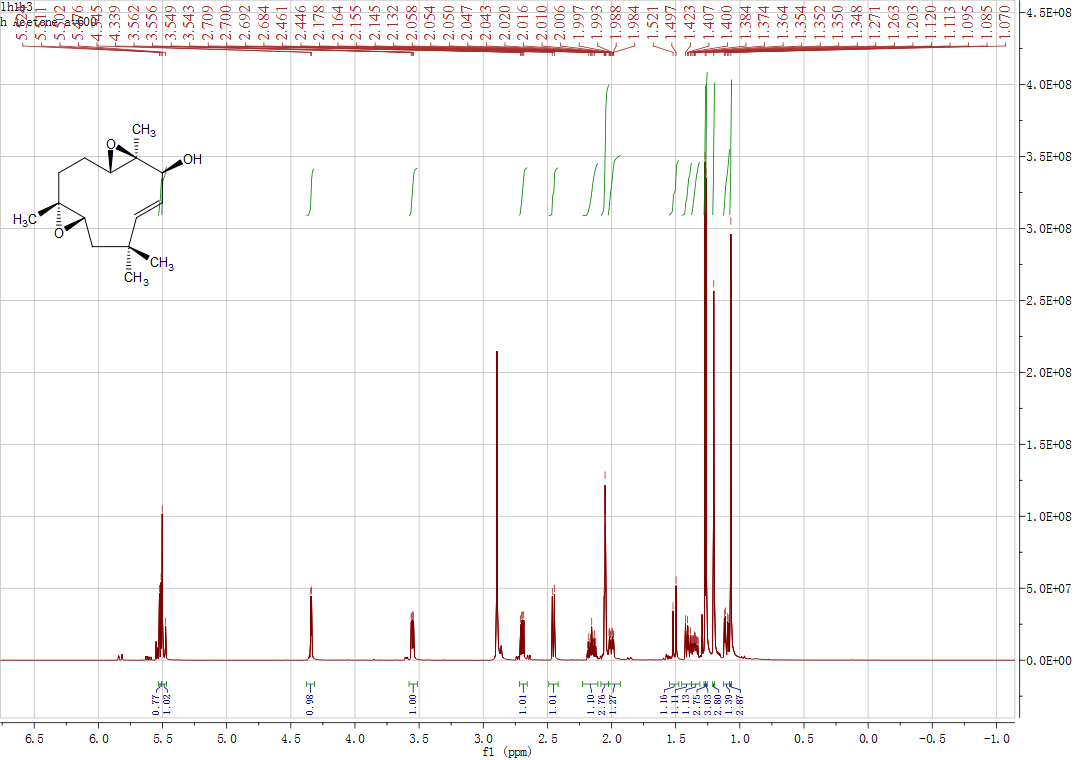


Figure 16S. ^13^C NMR and DEPT (150 MHz, acetone-*d*_6_) spectra of antrodol C (**3**)


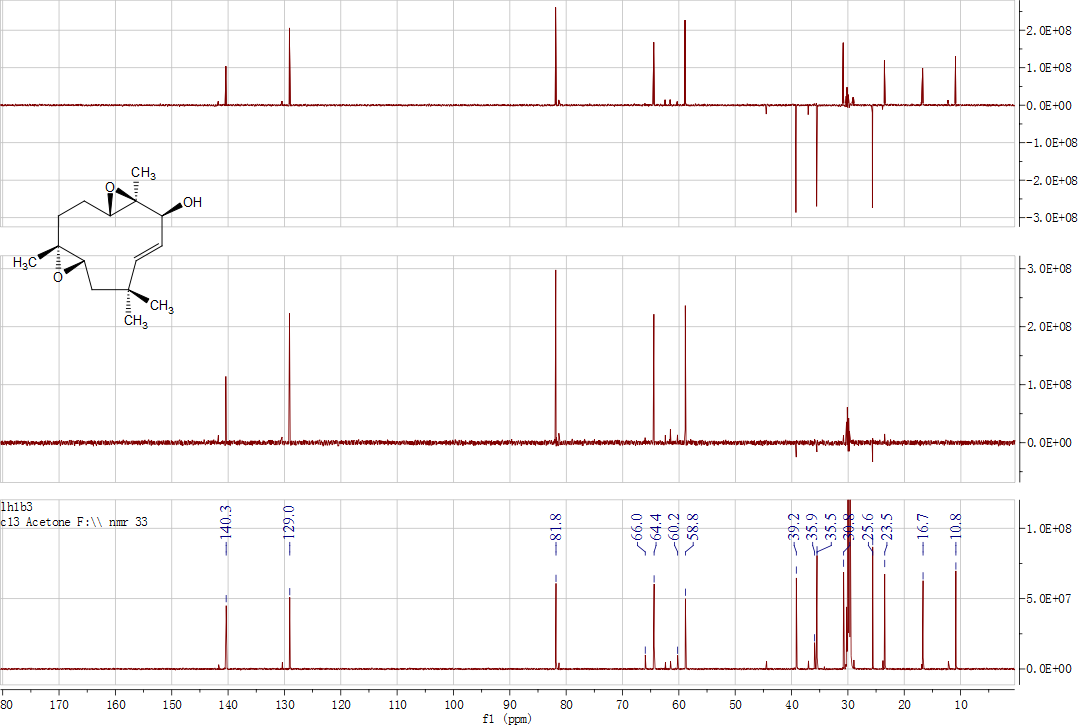


Figure 17S. ^1^H-^1^H COSY spectrum of antrodol C (**3**)


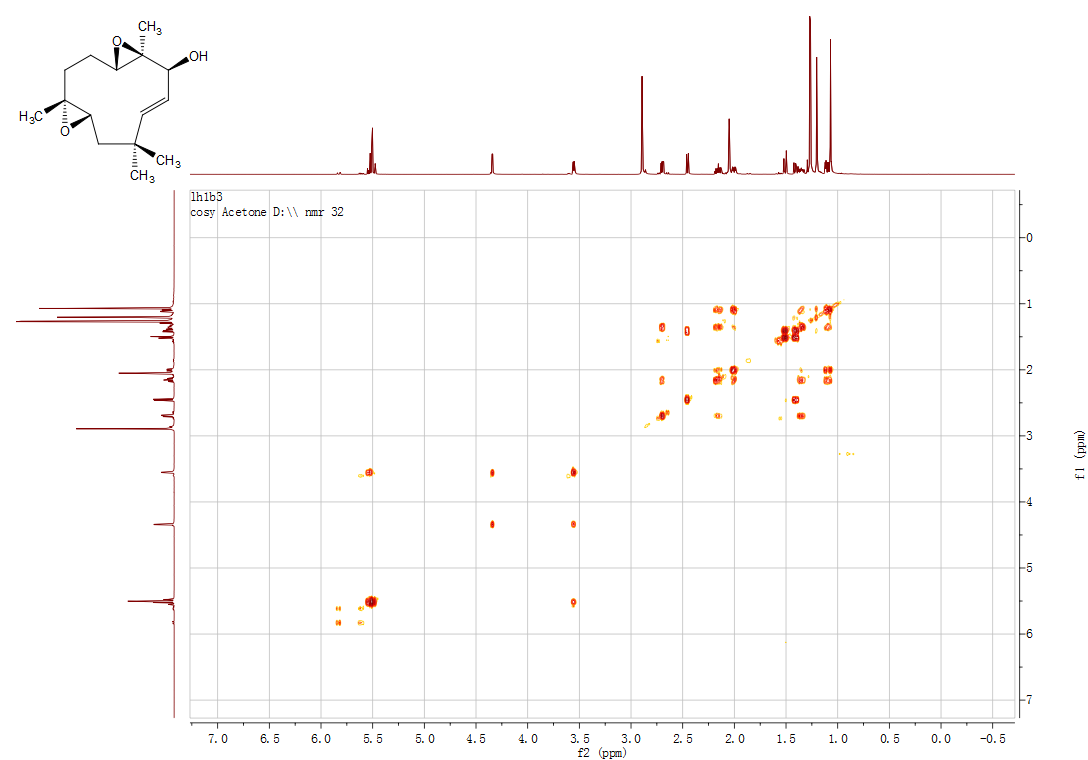


Figure 18S. HSQC spectrum of antrodol C (**3**)


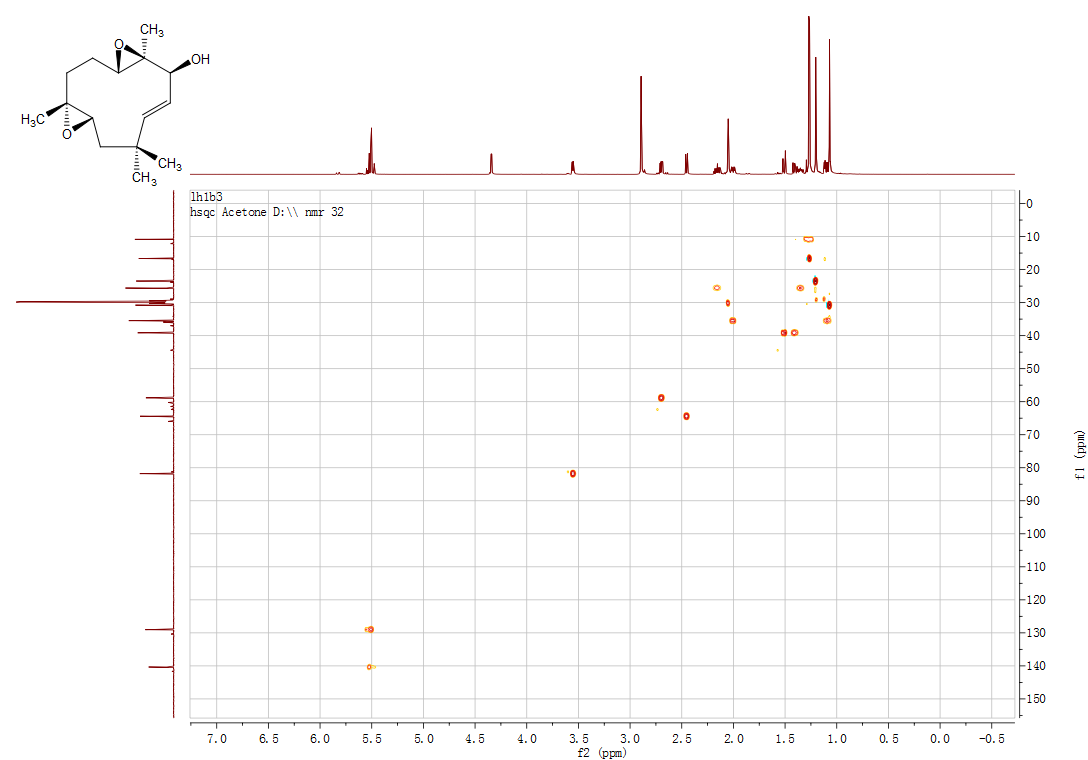


Figure 19S. HMBC spectrum of antrodol C (**3**)


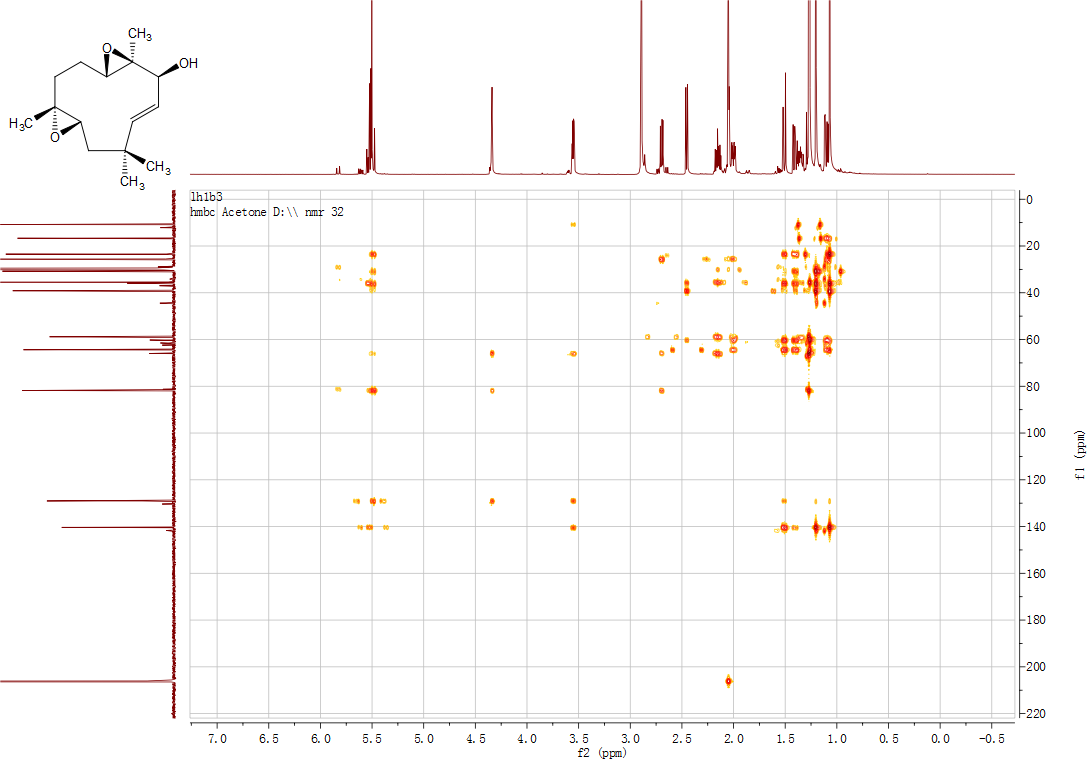


Figure 20S. ROESY spectrum of antrodol C (**3**)


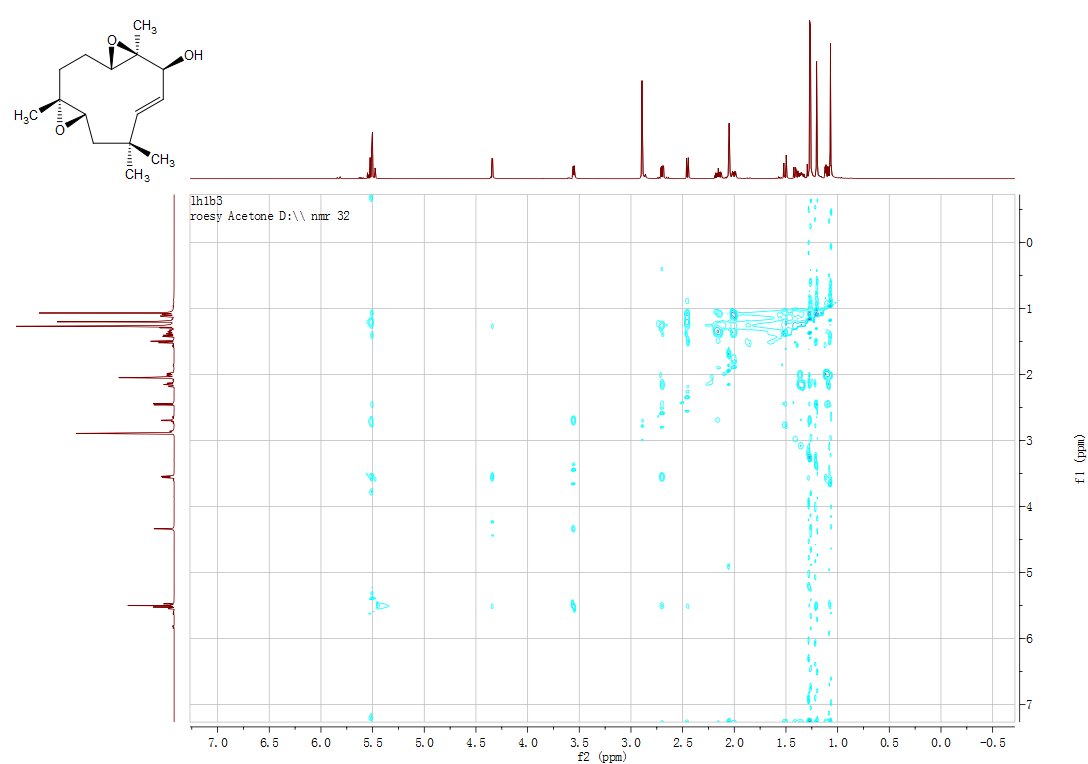


Figure 21S. HREIMS of antrodol C (**3**)


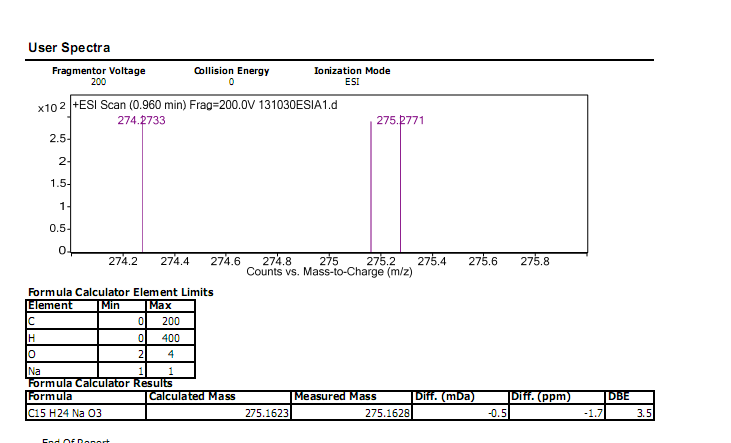


1. *Corresponding author at: 132# Lanhei Road, Kunming 650201, Yunnan, PR China. Tel.: +86 871 65216327; fax: +86 871 65212285.E-mail address: jkliu@mail.kib.ac.cn (J.K. Liu) [↑](#footnote-ref-2)
